# Structures of New World mammarenavirus glycoproteins as targets for antibody-mediated neutralization

**DOI:** 10.1128/jvi.01626-25

**Published:** 2026-05-27

**Authors:** Lily J. Taylor, Gustavo Helguera, Junki Maruyama, Jose A. Rodriguez

**Affiliations:** 1 Department of Chemistry and Biochemistry, University of California, Los Angeles (UCLA), Los Angeles, California, USA; 2 UCLA-DOE Institute for Genomics and Proteomics, University of California, Los Angeles (UCLA), Los Angeles, California, USA; 3 STROBE, NSF Science and Technology Center, University of California, Los Angeles (UCLA), Los Angeles, California, USA; 4 Laboratory of Pharmaceutical Biotechnology, Instituto de Biología y Medicina Experimental (IBYME-CONICET) 62921 , Buenos Aires, Argentina; 5 Department of Pathology, The University of Texas Medical Branch 198642, Galveston, Texas, USA; 6 Institute for Human Infections and Immunity, The University of Texas Medical Branch 551582, Galveston, Texas, USA; Indiana University Bloomington, Bloomington, Indiana, USA

**Keywords:** arenavirus structure, glycoprotein, neutralizing antibody, transferrin receptor

## Abstract

New World mammarenaviruses (NWMs) are viral pathogens endemic to the Americas, where several have acquired the capability for zoonotic transmission, leading to sporadic but potentially lethal viral hemorrhagic fever in humans. Several NWMs are classified as category A pathogens and are considered a persistent threat to human health due to their high case mortality rates, ability to spread through airborne transmission, and limited therapeutic and treatment options against them. Infection is mediated by the tripartite glycoprotein complex (GPC), which is the sole protein expressed on the viral envelope. The GPC, consisting of a receptor-binding domain (GP1), a fusion-enabling domain (GP2), and a stable signal peptide (SSP), governs viral entry initiated through the transferrin receptor 1 (TfR1). Subtle changes in GPC sequence and structure can drastically influence host range, immune recognition, and viral fitness, underscoring its central role in arenavirus pathogenicity and making it a prime target for therapeutic intervention. Over the past decade, advances in structural biology have enabled the elucidation of the molecular architecture of the NWM GPC and its interaction with host receptors and neutralizing antibodies. In this review, we discuss the information gleaned from structural interrogation of the NWM GPC and the significance of these findings on current therapeutic development against these lethal pathogens.

## THE NEW WORLD MAMMARENAVIRUS GLYCOPROTEIN COMPLEX

**Fig 1:**
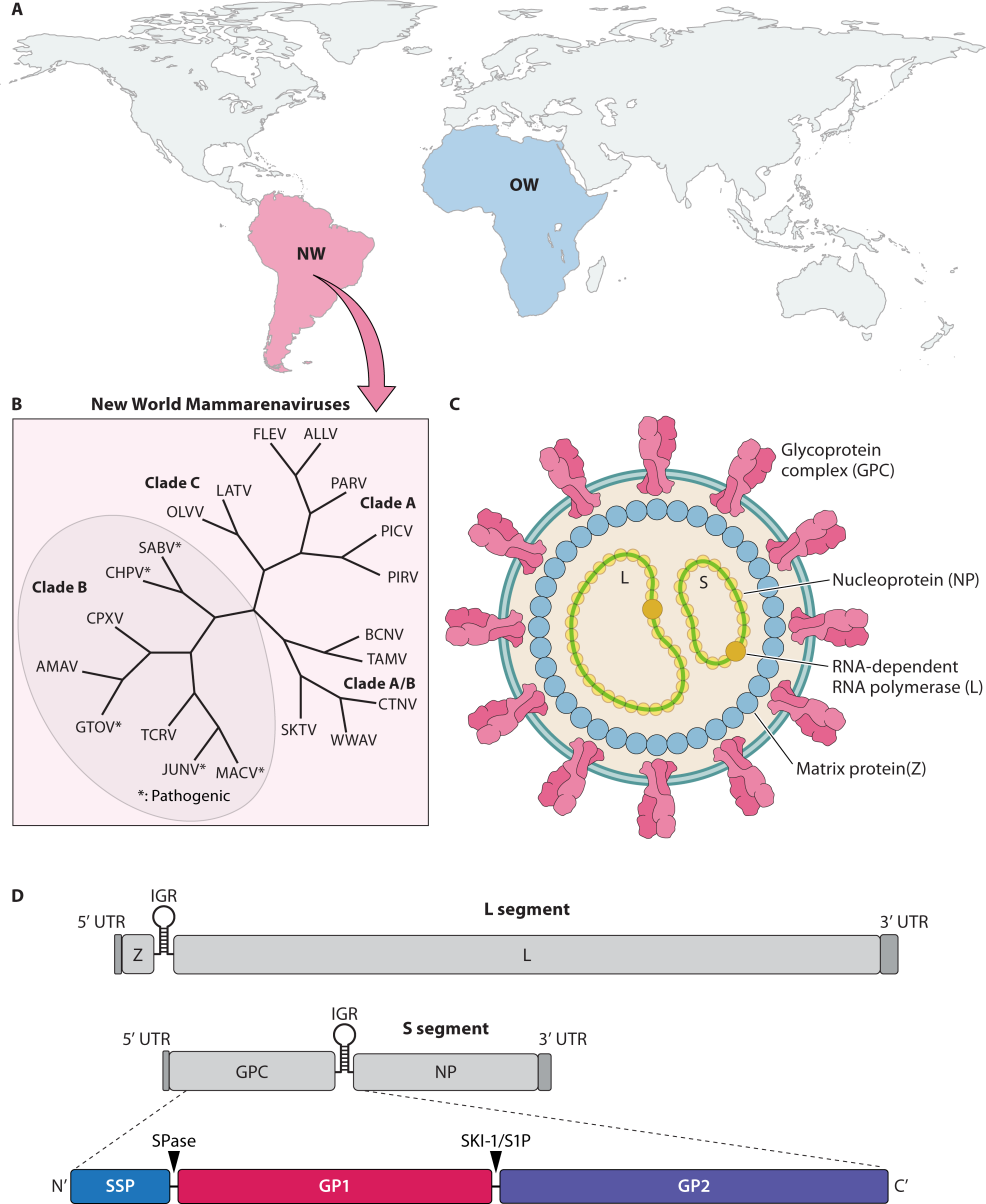
The New World mammarenaviruses and their surface glycoprotein complex (GPC). (**A**) Pathogenic New World viruses are primarily found in South America, where clade B species (**B**) have been associated with lethal outbreaks. Pathogenic species in the tree are noted with an asterisk (*) (5). (**C**) The viruses are enveloped and display a glycoprotein complex (GPC) on their surface; they encapsulate a matrix protein (Z), nucleoprotein (NP), and two ambisense RNA transcripts, L and S. (**D**) The L transcript encodes an RNA-dependent RNA polymerase and the matrix protein, while the S transcript encodes the nucleoprotein and glycoprotein complex precursor. The latter is cleaved at two sites by proteases, SPase and S1P, to produce three polypeptides: a stable signal peptide (SSP), a receptor-binding domain (GP1), and a transmembrane fusion domain (GP2).

Mammalian arenaviruses (mammarenaviruses) are enveloped, bisegmented RNA viruses that are broadly divided into two evolutionary subgroups based on their phylogeny and geographic distribution: Old World (OW) and New World (NW) (Fig. 1A) (1, 2). Old world mammarenaviruses (OWMs) include the Lassa virus (LASV), which is endemic to West Africa, as well as the lymphocytic choriomeningitis virus (LCMV), which is more broadly distributed (3). New World mammarenaviruses (NWMs) are endemic to South America and are further subdivided into clades A, B, A/B, and C. (4). Among these, clade B includes most of the known pathogenic NWMs (Fig. 1B) (2).

Mammarenaviruses primarily circulate in rodent reservoir hosts where their infection is asymptomatic, but several OW and NW viruses have demonstrated the ability to transmit zoonotically to humans, where infection can result in severe and potentially fatal hemorrhagic fevers (6). Despite their ecological and evolutionary divergence OW and NW arenaviruses share a conserved genomic architecture. Their bisegmented ambisense genome encodes only four proteins, with the large (L) segment encoding the Z matrix protein and the RNA-dependent RNA polymerase, and the small (S) segment encoding the surface glycoprotein complex (GPC) and the nucleoprotein (Fig. 1C and D) (2).

**Fig 2:**
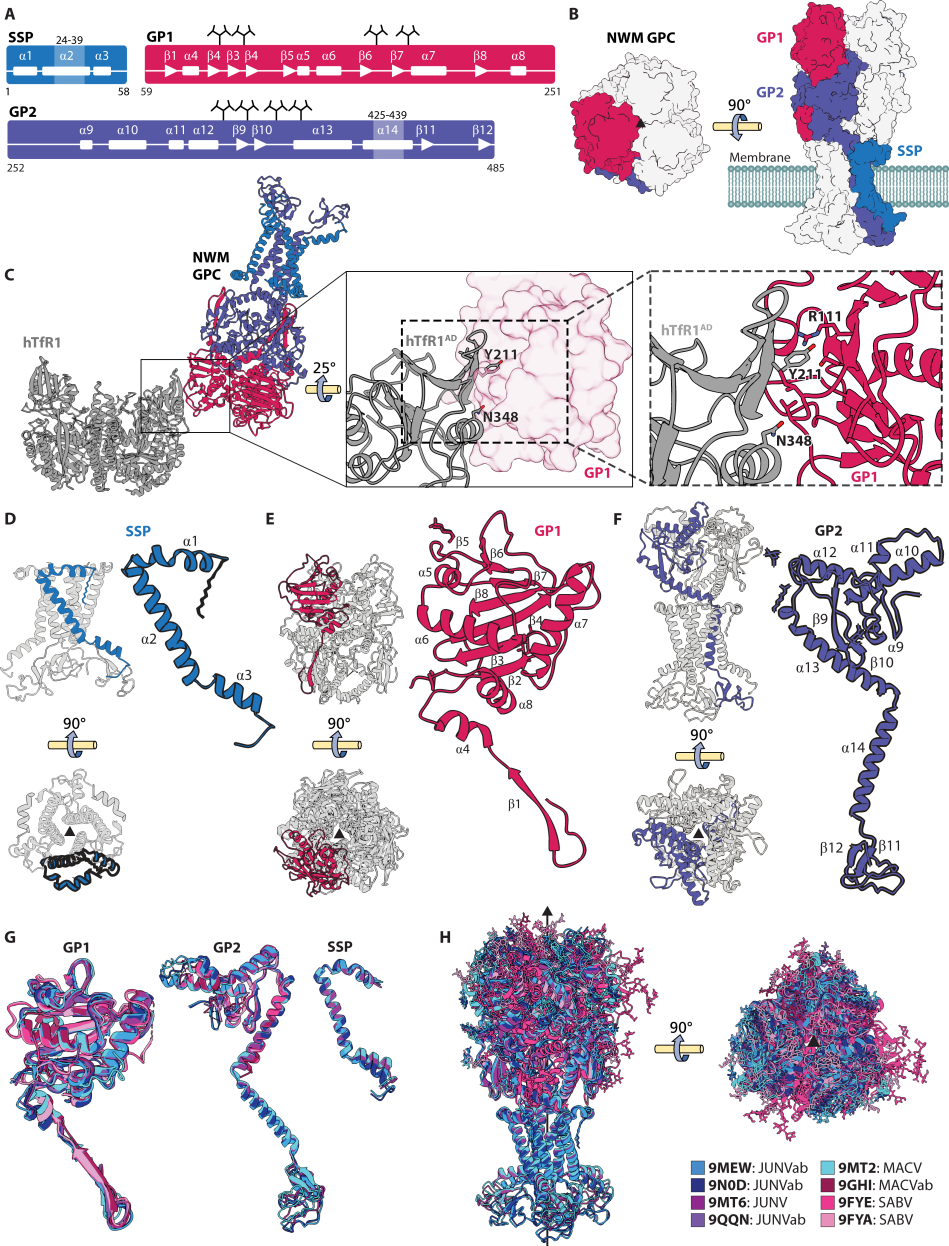
Structural snapshots of the mammarenavirus GPC and its subcomponents. (**A**) Secondary structure features and glycosylation sites of the SSP, GP1 and GP2 polypeptides of the JUNV GPC. (**B**) Top and side views of a threefold symmetric, membrane-embedded, mature GPC assembly; the components of one-third of the complex are colored: GP1 (pink), GP2 (purple), SSP (blue). A triangle indicates the axis of symmetry. (**C**) A docked model of one mature GPC onto its binding site on hTfR1 shows its apical domain (hTfR1^AD^) engaging the receptor-binding cleft of GP1; insets illustrate structural features of the GP1-hTfR1^AD^ interaction. Side and top views of the structures of SSP (**D**), GP1 (**E**), and GP2 (**F**) within their threefold symmetric assemblies. Subcomponents from the structures of nine full-length GPCs aligned per subunit (**G**) show conserved geometries. (**H**) The full GPCs superimposed and aligned by their transmembrane domains show considerable interdomain rearrangement, despite a retained threefold symmetry in the complex.

Among these viral components, the surface GPC is principally responsible for mediating viral entry through receptor engagement and membrane fusion, thereby defining host tropism and pathogenic potential (7). That potential is correlated to the ability of the GPC to bind specific host receptors, such as the transferrin receptor 1 (TfR1) in the case of clade B NWMs (8). Although some NWMs can enter cells independent of TfR1, their internalization via clathrin-mediated endocytosis highlights the importance of interactions with co-receptors or other molecules at the cell surface that may influence both pathogenicity and disease presentation (5, 8–10). The receptor dependence and host tropism of arenaviruses are therefore dictated by the GPC, which is initially expressed as a single glycoprotein precursor and is cleaved by host cell proteases, signal peptidase, and subtilisin kexin isoenzyme 1 into the receptor-binding domain glycoprotein 1 (GP1), the fusion-enabling domain glycoprotein 2 (GP2), and the stable signal peptide (SSP) (Fig. 2A) (11). Together, these components form a class 1 viral fusion machinery that coordinates receptor binding, cellular entry, and pH-induced viral fusion leading to infection (8).

OWMs and NWMs differ most substantially in their receptor usage, which is dictated by their GPC. While OWMs and clade C NWMs rely on α-dystroglycan to enter cells (12), non-clade C NWMs use the iron-trafficking transferrin receptor 1 (TfR1) (8). It is noteworthy that, while TfR1 is considered the primary cellular receptor for NWMs and all pathogenic NWMs engage human TfR1 (hTfR1) to initiate infection(13), there is evidence to suggest NWM GPC-mediated entry through TfR1-independent pathways (8). Nonetheless, several clade B NWMs, including Junin virus (JUNV), Machupo virus (MACV), Sabia virus (SABV), Guanarito virus (GTOV), and Chapare virus (CHAPV), rely on a conserved site on the apical domain of hTfR1 to enable human infection (14).

## NWM GPC FORM AND FUNCTION

The NWM GPC exhibits high sequence diversity while retaining its functional role as a mediator of attachment to host cells and of membrane fusion. This diversity is reflective of selective pressures imposed by differences in host receptor usage, geographic range of natural reservoirs (Fig. 1A and B), and immune environments, while remaining constrained by the requirement to assemble into a trimeric (Fig. 2B), metastable, and dynamic prefusion complex capable of undergoing the conformational changes associated with pH-induced viral fusion.

Due to the variable selective pressures acting on the GPC, its sequence variation is unevenly distributed across its subunits. The receptor-binding domain, GP1, exhibits high sequence variability, reflective of its adaptation to distinct host receptors and its role in evading host immune factors. Mutational studies have confirmed that each NW GP1 shows specific, high affinity for its host receptor (7), though all pathogenic NWM GP1s are expected to contact hTfR1 at a fixed interface. That interaction involves a prominent loop on the hTfR1 apical domain (hTfR1^AD^) which includes Tyr211, a residue common to TfR1 orthologs of pathogenic NWM hosts (Fig. 2C) (7). Despite this shared mode of engagement, GP1s display high variability at this interface, owing both to residue differences and to insertions/deletions which alter specific GP1-hTfR1 contacts across pathogenic NWMs (14). These findings are consistent with the understanding that GP1 sequence variation has been shaped by long-standing co-evolution within its host species (15), ultimately leading to GP1-hTfR1 compatibility arising from the natural evolution of NW arenaviruses with their natural hosts and dictating their pathogenicity (16).

In contrast, GP2 and the SSP maintain highly conserved sequences, reflecting their tightly regulated role in facilitating viral fusion. Key structural elements are required to enable its class 1 fusion mechanism, and this region is more shielded from host immune factors. However, sequence variation within the GP1 that has been acquired to optimize receptor contacts tends to accompany complementary changes in GP2 in order to preserve interdomain contacts and fusion capabilities. This is evidenced by the deleterious effect of domain swap chimeras when assessed on the surface of pseudovirions (17, 18).

## AN EVOLVING STRUCTURAL UNDERSTANDING OF NWM GPCs

A structural understanding of the NWM GPC and its components began with early biochemical and NMR studies which identified key roles for GP1, GP2, and the SSP in receptor binding and membrane fusion and their proposed topology within the viral membrane (19, 20). However, a complete understanding of how these components assembled into a functional complex was lacking, as was the full impact of sequence variation across the GPC on altered receptor-binding capabilities and immune recognition. Structural interrogation of the whole GPC has complemented decades of biochemical and molecular work to answer these questions. Persistent structural biology efforts have now transformed our understanding of the NWM GPC.

Structural information on OWM and NWM GPCs has expanded dramatically due to advances in X-ray crystallography and cryo-electron microscopy (cryoEM). Early structural studies, which focused on isolated GPC components, provided high-resolution insights into the GPC’s domain architecture and interactions with key host and immune factors, guiding subsequent efforts on full-length GPC characterization. These have revealed the highly structurally conserved architecture of the GPC and its components (Fig. 2D through G).

Initial crystallographic studies of isolated GP1 domains from both OW and NW arenaviruses revealed a primarily conserved architecture for the receptor-binding domain despite substantial sequence divergence and distinct host receptor usage (21, 22). These early structures further clarified how the NW GP1 engages hTfR1 to initiate infection (14) and revealed key determinants of receptor compatibility, principles that were also observed in structural studies of the NW GP1 in complex with neutralizing antibodies (23, 24). Complementary structural characterization of isolated GP1 and GP2 segments confirmed the class 1 fusion protein architecture of the GPC and began to define the molecular basis for GPC-mediated membrane fusion (25–27).

The application of cryoEM to arenavirus glycoproteins marked a major turning point in understanding the role of the GPC in viral entry. The first stabilized, prefusion structures of OWM GPCs revealed the trimeric organization of the complex, with GP1 receptor-binding domains positioned atop a GP2 domain-swapped core and the close association of the SSP forming a six-helix bundle with GP2 within the viral membrane (28, 29). These structures highlighted the unusual role of the SSP in stabilizing the trimeric assembly and regulating viral fusion activity. Subsequent cryoEM structures of NWM GPCs from MACV, JUNV, and SABV viruses demonstrated a broadly conserved prefusion architecture consistent with a class 1 fusion complex while revealing distinct GP1 orientations and interdomain contacts that reflect differences in receptor engagement and host specificity (Fig. 2G and H) (30, 31). The functional importance of this architecture is further underscored by cryoEM structures of full-length, natively embedded NW GPC structures, which reveal the tight association of the SSP within the lipid membrane and the luminal portion of the GPC (32). Implicitly encoded into the growing collection of GPC structures is a sense of the dynamics of the complex. An alignment of GPC subunits from structures determined to date shows high structural similarity, while the whole complexes aligned at the membrane display an array of distinct conformations and orientations at their apex (Fig. 2G and H).

**Fig 3:**
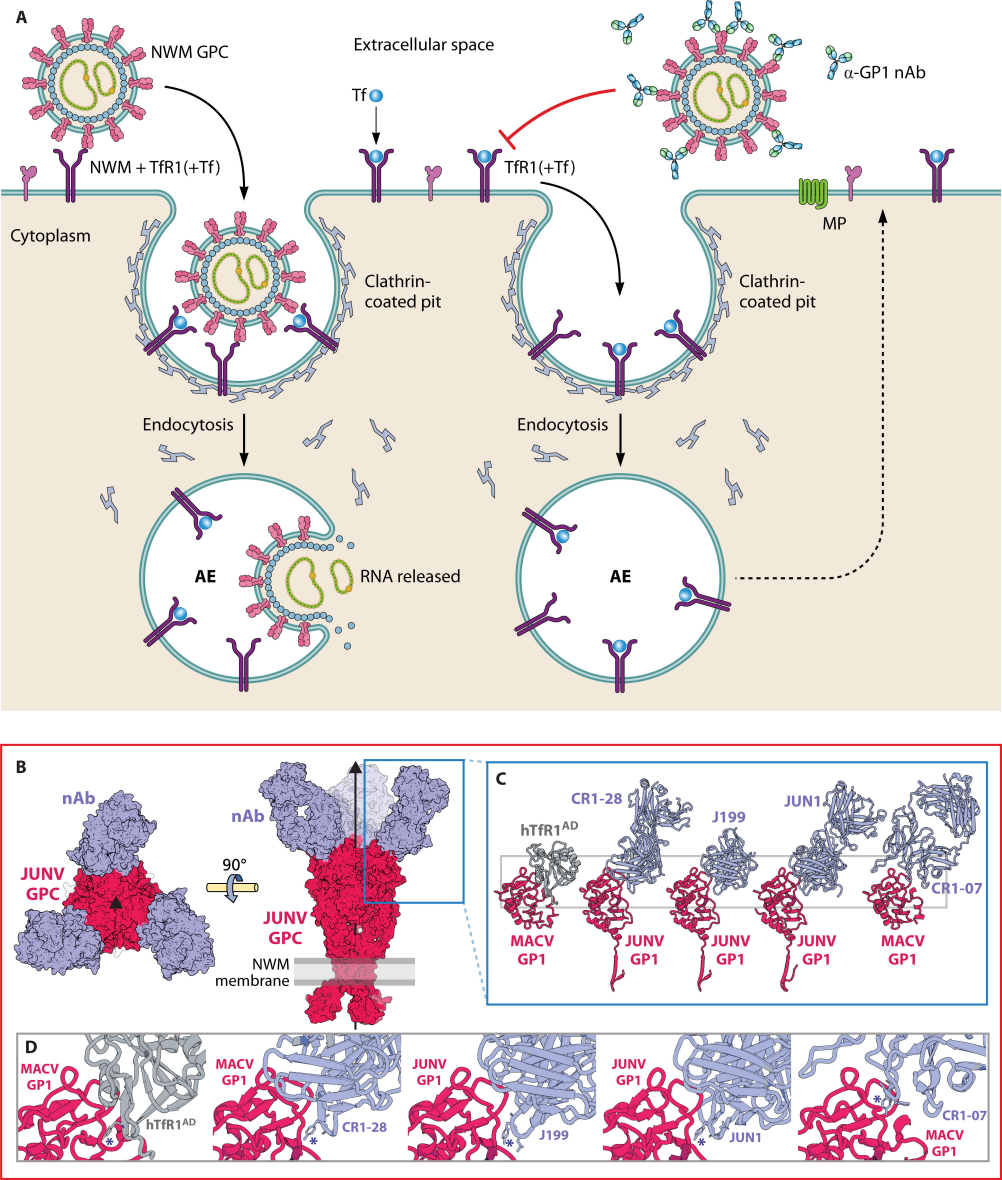
Inhibition of cellular uptake of NWMs by GPC-neutralizing antibodies. (**A**) Schematic model of the interaction between GPC and hTfR1 that facilitates clathrin-mediated (CCP) uptake of NWMs into the endosomal trafficking network and ultimately acidified endosomes (AE), where conformational GPC changes enable fusion. NWM GPCs are seen to interact with hTfR1 irrespective of its binding to Tf. Neutralizing antibodies (α-GP1) interacting with the GPC preclude its binding to hTfR1 and cellular uptake of NWMs. (**B**) Red inset shows top and side views of the membrane-embedded JUNV GPC bound by a neutralizing antibody. Arrows indicate its axis of threefold symmetry. (**C**) Blue inset shows structurally similar interactions between hTfR1^AD^ or neutralizing antibodies, CR1-28, J199, JUN1, CR1-07, and the JUNV or MACV GP1s. The antiviral arenacept is expected to interact like the hTfR1^AD^ (37). (**D**) Gray inset shows magnified views of GP1 complexes in panel C. An asterisk (*) indicates hTfR1 residue Y^211^ and key residues on bound antibody paratopes, including ligand-binding antibody tyrosines (23, 38, 39).

Beyond prefusion structures, cryoEM analyses of GPCs in complex with neutralizing antibodies have also provided critical insights into the mechanisms of immune recognition and neutralization (Fig. 3A). Several potent antibodies target epitopes proximal to the receptor-binding interface, supporting the functional importance of this region in both viral entry and viral neutralization (Fig. 3B and C) (29, 32, 33). Additional modes of neutralization have been observed, including lateral antibody binding in OWMs (34, 35), although analogous mechanisms have yet to be structurally observed for NWMs. Recent cryoEM structures of GPCs embedded in intact viral particles have extended these findings to the native membrane context, revealing the spatial organization and conformational state of the NWM GPC on the virion surface (32). Furthermore, structures of OWM GPCs under varied pH conditions have begun to capture the conformational heterogeneity associated with viral fusion activity (36).

## THE NWM GPC AS A TARGET FOR NEUTRALIZING THERAPIES AND THE PROSPECT OF STRUCTURE-GUIDED NWM VACCINES

The early development of antibodies targeting NWMs demonstrated the challenges in generating broad neutralizing activity. Shortly after the discovery of the NWMs and their structural components, their antigenic recognition illustrated the close relationship between lethal clade B agents, compared to their distant cousins LCMV and LASV (40). Studies also revealed that immunization could elicit both highly specific, neutralizing antibodies against GPC, as well as GP2-specific or nucleoprotein-targeting antibodies. The latter were more broadly cross-reactive due to higher conservation of the nucleoprotein relative to the GPC and, in certain cases, could also recognize OWMs (40, 41). In surprising cases, antibodies directed not at the GPC but at the nucleoprotein have also been reported to neutralize attenuated NWM strains in a proposed mechanism by preventing viral replication by disrupting viral entry at the cell surface (42), but there is currently no further evidence for participation of the nucleoprotein in viral entry, and neutralization has principally required targeting of the GPC and, in particular, GP1 (23, 24, 38). This pattern has been established by panels of monoclonal antibodies raised against specific strains of a species, like JUNV, which gave rise to species-specific neutralizers with high titers. One of those, GB03-BE08 (J199), would later prove highly effective, as a recombinant chimera, at mitigating JUNV infection in a guinea pig model of Argentine hemorrhagic fever (AHF) (43). Such successful GPC-oriented single-agent neutralizing antibodies have been and continue to be actively developed against other pathogenic NWMs, including MACV (44). This has resulted in a growing list of neutralizing antibodies specific for pathogenic NWMs, and offered opportunities to assess the structural basis for their specificity and mechanism of neutralization (Fig. 3).

The ability to recombinantly produce and structurally investigate intact GPCs or their subcomponents, has yielded detailed structural information on antibody-bound complexes, improving our understanding of the convergent mechanisms of antibody-mediated GPC neutralization (38). Whether derived from immunization with intact virions or structural NWM components, the neutralizing antibodies shown to target GP1 do so in a manner that obstructs its receptor binding (23, 24). Crystallographic structures of neutralizing antibodies bound to isolated GP1 have shown conserved paratope motifs that engage the receptor footprint on GP1 in a manner that mimics receptor binding (14). The details of this interaction involve the insertion of a tyrosine residue into the receptor-binding cleft of GP1, a mechanism true for antibodies JUN1 (24), OD01 (24), GD01 (24), and CR1-28 (23, 24). The structure of CR1-28 bound to full-length, membrane-embedded GPC shows a shared binding mode that closely aligns with its isolated GP1-bound state; it also aligns with the GPC-bound state of J199 (32). Intriguingly, the epitope of the JUNV-MACV cross-neutralizing antibody, CR1-07 is distinct from that of the JUNV-specific neutralizing antibodies (23). Its footprint is shifted on GP1 to accommodate an 11-residue loop insert present in the MACV sequence, a loop that has been reported to specifically impede GP1 recognition by JUNV-neutralizing antibodies (17). While the CR1-07 epitope is distinct (Fig. 3C), it overlaps with other MACV-specific neutralizing antibodies, which also bind an epitope displaced from that of other JUNV neutralizing antibodies (24). Perhaps unsurprisingly, our collective knowledge of neutralization-competent epitopes on GP1 point to its receptor-binding site as a key target (23, 24). This is supported by studies that have shown neutralizing antibodies in the plasma of JUNV convalescent patients pointing to the steric occlusion of receptor binding as an effective defense mechanism against AHF (39), suggesting that antibody-based neutralization approaches could succeed against other lethal NWMs (7, 24, 44, 45). This therapeutic approach is, however, not successful in treating Lassa fever, potentially due in part to differences in the glycan density on the GPC surface (46). In general, N-linked glycosylation motifs across the GPC challenge the ability of bound nAbs to effectively neutralize NWMs, which variability decorate across OWM and NWM GPCs (Fig. 2H) (46).

**Fig 4:**
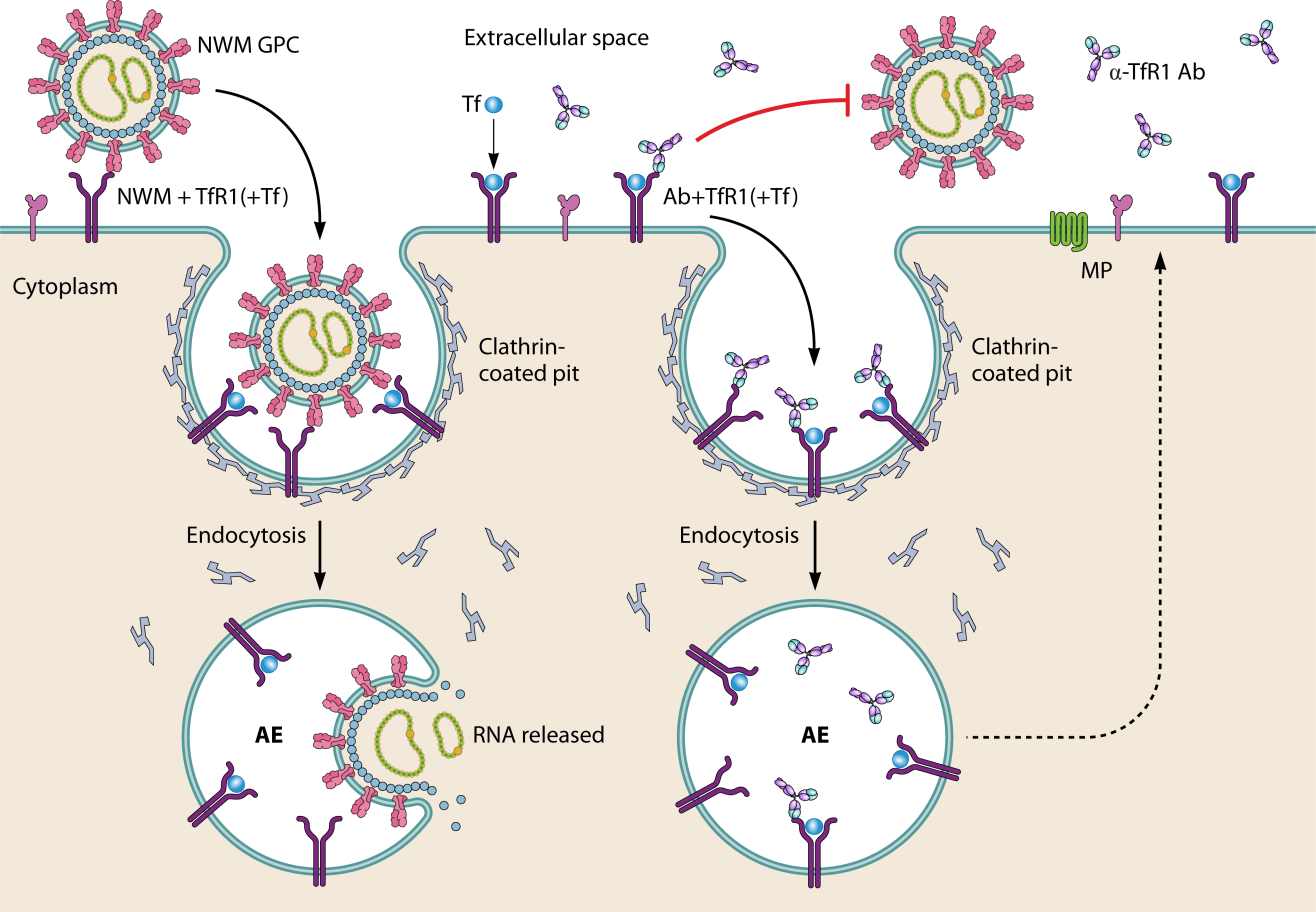
Inhibition of cellular uptake of NWMs by host-directed broadly neutralizing antibodies. A schematic shows hTfR1-mediated uptake of NWMs into clathrin-coated pits and trafficking to acidified endosomes (AEs), where conformational GPC changes enable fusion. Host hTfR1-directed antibodies are shown precluding receptor engagement by GPC and instead facilitating their hTfR1-mediated uptake and recycling, irrespective of its binding to Tf.

Another validation of the value of hTfR1 blockade as a viable solution to NWM infections comes from host-directed therapies (47). While various receptors and co-receptors have been suggested to enable cellular internalization of NWMs, blockade of the hTfR1-GPC interaction remains principally associated with effective NWM neutralization (8, 10, 11, 17, 39, 44). hTfR1 engagement has been exploited by both receptor-mimicking antivirals (37, 48) and receptor-targeting antibodies that recognize the NWM binding site (Fig. 3 and 4). The latter have demonstrated the ability to mitigate lethal JUNV infection in animal models and broadly prevent internalization by pathogenic clade B agents in an apical domain-dependent manner (47, 49–51). Remarkably, 80% of hTfR1-expressing mice treated with a mouse-human chimeric anti-hTfR1 IgG1 variant with impaired FcγR and C1q binding survived challenge with JUNV (49), demonstrating the opportunity for potent host-directed treatment against NWM infections (Fig. 4).

As knowledge of GPC structure and function, its targeting, and receptor engagement grows, the potential for eliciting broadly neutralizing anti-GPC responses increases. Recent efforts with chimeric and multivalent GPC vaccines (52) further the long-standing promise of an effective live attenuated NWM vaccine, exemplified by Candid#1 (53, 54). Key to realizing this promise may be structure-guided vaccine efforts (55). Structures could directly inform on target epitopes (32) and prefusion-stabilized GPC states (30) and open the door to machine learning-assisted immunogen or antibody design, as has been demonstrated for other challenging vaccine targets (56, 57). These, in turn, could overcome the sequence divergence in pathogenic NWM GP1s that shape the rugged landscape challenging broadly neutralizing immune responses.

## CONCLUSIONS AND OUTLOOK

A growing collection of structural snapshots of NWM GPCs is furthering a more complete understanding of their molecular mechanisms to be exploited for antiviral therapy. Antibody-mediated neutralization of NWMs has already been robustly achieved against single agents. Their structures and the structural basis for some cross-neutralizers are broadening our understanding of receptor blockade mechanisms. Congruence between the mechanism of neutralization by immunized or convalescent patient polyclonal sera and species-selective or cross-reactive neutralizing antibodies supports receptor blockade as a key GPC vulnerability. It also presents avenues for the further development of pan-clade B NWM neutralizing antibodies and/or host-directed agents inhibiting NWM infection.

As structural insights into GPC structure and function deepen, the impact of GPC dynamics and its glycan shield on neutralizing antibodies presents opportunities for further investigation. Studies have suggested that the pattern of decorating glycans plays a role in antibody recognition and ultimately neutralization of GPCs, directly impacting the kinetic parameters of antibody binding, their occupancy, and capacity for virus neutralization (46, 58). For example, immunization with partially glycan-deficient GP1 variants robustly generated neutralizing antibodies that were largely specific for the glycan-deficient variants; the latter were generally more readily neutralized (46). In fact, the occupancy of N-linked glycans at GPC N83 and N166 in MACV has been directly shown to correlate with pathogenicity that is in turn linked to neutralizing antibody susceptibility *in vivo* but not fitness in cell culture (59). The role of GPC dynamics, both at the receptor-binding domain and at the membrane, is likewise in need of investigation. The pH-driven conformational changes that drive fusion in acidified endosomal compartments have been linked to networks of GP2 and SSP residues and, in particular, membrane proximal residues like the pH-sensing SSP residue K33 (30, 60). Biochemical studies have shown that mutations at position 33 in the SSP alter the pH necessary to activate membrane fusion, and K33 may destabilize the GPC at low pH, contributing to the initiation of fusion events (60). These sites are also targets of arenavirus-specific fusion inhibitors that have the potential to impact fusion-associated conformations, as illustrated by recent structures of the pH-induced conformational landscape of the LASV GPC and its structure bound to the small molecule fusion inhibitor ARN-75039 (36). Similar dynamics are expected for NWM GPCs but have yet to be elucidated; we await the structures of pH-altered NWM GPC conformations and glimpses of their interaction with antibodies or small molecule inhibitors.
